# Effects of erythropoietin for precaution of steroid-induced femoral head necrosis in rats

**DOI:** 10.1186/s12891-018-2208-2

**Published:** 2018-08-07

**Authors:** Yong-Qing Yan, Qing-Jiang Pang, Ren-Jie Xu

**Affiliations:** 1 Department of Orthopaedics, Ningbo No.2 Hospital, Xibei Street No.41 Ningbo, 315010 Zhejiang, People’s Republic of China; 2 Department of Orthopaedics, Suzhou Municipal Hospital/The Affiliated Hospital of Nanjing Medical University, No 26, Daoqian Street, Suzhou, 215000 Jiangsu People’s Republic of China; 3 Department of Orthopaedics, the First Affiliated Hospital, Orthopaedic Institute, Soochow University, Suzhou, 215000 Jiangsu People’s Republic of China

**Keywords:** Erythropoietin, Steroid, Necrosis, Rat, Femoral head

## Abstract

**Background:**

Steroids such as glucocorticoid have been widely used for their excellent anti-inflammatory, anti-immune, and anti-shock properties. However, the long-term use in high doses has been found to cause necrosis of femoral head and other serious adverse reactions. Thus, it is of great importance to safely use these medications on patients without inducing bone necrosis.

**Methods:**

In this preclinical study, we examined the effects of erythropoietin (EPO) to attenuate the induction of steroid-induced femoral bone necrosis using rats to build up the in-vivo models. Rats were randomly divided into three groups: negative control group (group A), disease group (group B), and EPO group (group C). 20 mg/kg methylprednisolone was administrated into group B and group C for 6 weeks with two intramuscular injections per week per rat. Group C was further given daily intraperitoneal injections of rHuEPO during this period. Group A received only injection of saline at the same schedule. 12 weeks after the initial drug administration, the rats’ femoral tissues were harvested for HE staining, immunohistochemistry studies for PECAM-1(also CD31) expression and Western Blotting for VEGF expression.

**Results:**

Histology studies showed that compared with the disease group, EPO group had significant improvement and bone morphology being much closer to the negative control group. Immunohistochemical studies revealed that EPO group had statistically much more expression of PECAM-1 than the other groups did. Western Blot demonstrated that the EPO group had significantly higher VEGF expression than the disease group.

**Conclusion:**

Results suggested that simultaneous injection of EPO could partially prevent steroid-induced ANFH.

## Background

Osteonecrosis is often observed in hips, knees, shoulders, and ankles, in which avascular necrosis of the femoral head (ANFH) is the most common one. Approximately 80% of patients suffering from ANFH progress to a collapse of the femoral head, resulting in impaired hip joint function and permanent disability if did not receive appropriate treatments [1].

Glucocorticoid (GC) administration is the most common non-traumatic cause of ANFH [2]. About 25% of patients receiving hormone therapy would eventually develop osteonecrosis [3], therefore early prevention is of most importance. However, the exact pathogenesis remains to be fully elucidated. There are several hypotheses including fat embolization, intramedullary pressure changes, coagulation disorders, circulatory impairment and cell dysfunction and apoptosis [4–6]. Amongst these mechanisms, the vascular hypothesis, in which local microvascular impairment leads to a decrease in blood flow in the femoral head, has become more widely accepted [7–11].

Erythropoietin (EPO) is a glycoprotein excreted by the kidneys. Its main function is to stimulate the proliferation and differentiation of reticulocytes. Further studies proved that erythropoietin could also promote angiogenesis [12]. Studies have shown that erythropoietin is a multifunctional factor that exerts extensive protective effects in a variety of non-hematopoietic organs [13–16], which can promote cell regeneration and vessel formation; resist inflammation, oxidation, and apoptosis; and accelerate vessel formation, cell proliferation, and cell protection [17–19]. Recently, erythropoietin has been reported to be capable of promoting bone healing [20]. To summarize, apoptosis of bone cells, microcirculation impairment and cell dysfunction could be possible mechanisms in ANFH. And EPO can inhibit cell apoptosis promoting angiogenesis and proliferation which might counteract side effect of GCs. Therefore, we hypothesize that EPO might have some positive effects on preventing steroid-induced ANFH.

## Methods

### Materials

Human erythropoietin (10,000 U, Shenyang Sunshine Pharmaceutical Co., Ltd. Shenyang, China); Goat polyclonal to PECAM-1 Antibody (1:50, Santa Cruz, CA, USA); Rabbit anti-sheep SP immunohistochemical staining kit and citrate antigen retrieval solution (pH 6.0) (Fuzhou Maixin Biotech. Co., Ltd., Fuzhou, China); Hematoxylin (Sigma, St. Louis, USA); PVDF membranes (Millipore Company, MA, USA); Polylysine solution (0.1%) and DAB Horseradish Peroxidase Color Development Kit (Beijing Golden Bridge Biotechnology Co., Ltd., Beijing, China); Prestained Color Protein Molecular Weight Marker (Fermentas, Waltham, MA, USA); Tris-Hcl/SDS solutions (1.5 mM, pH 8.8; and 0.5 mM, pH 6.8, Sangon Biotechnology, Shanghai, China); Acrylamide/Bis solution (30%, Bio-Rad, California, USA); BeyoECL Plus kit, SDS-PAGE Sample Loading Buffer (5×), Western blot, IP cell lysates, PMSF together with BCA protein assay kit (Beyotime Biotechnology, Shanghai, China); Skim milk powder (Yili Group, Inner Mongolia, China); VEGF Antibody (Abcam, Cambridge Science Park, UK); β-actin Antibody, goat anti-Rabbit IgG-HRP, and goat anti-Mouse IgG-HRP (Bioworld, Minnesota, USA).

Eighteen SD male rats and eighteen SD female rats were supplied by Hangzhou hi-biotechnology Co., Ltd. (Hangzhou, China).

### Animal models

The adoption of 36 rats in this study was approved by Institutional Animal Care and Use Committee and complied with NIH animal usage guidance. These eighteen male rats and eighteen female rats were randomly assigned into three groups (6 male rats and 6 female rats for each group) using a random number table: the negative control group, disease group, and EPO group. Methylprednisolone (20 mg/kg bodyweight) was muscularly intramuscularly injected into one of the hind legs of each rat from both group B and Group C twice a week for 6 weeks. Every rat from the Group C was further given a daily intraperitoneal injection of rHuEPO (500 u/d/kg-bodyweight) during these 6 weeks. The group A only received injection of saline at the same schedule. Rats were housed in cages together for 12 weeks (including 6 weeks after completing treatment) and had freely available food as well as water for the whole period.

The animals were sacrificed after 12 weeks. Under general anesthesia a lethal dose of pentobarbital (80 mg/kg BW) was injected. Femurs of both hind legs were dissected under sterile conditions and further investigations were conducted (72 femurs in total). Some harvested femoral tissues from each group were dehydrated accordingly and embedded in wax before being sliced into thin slices for HE stain (6 femurs for each group) and immunohistochemistry studies (12 femurs for each group). The rest samples (3 femurs for each group) were used for Western blot experiments.

### Histological examination

Some tissue slices embedded in wax from each rat were de-waxed using xylene and then gradually re-hydrated using ethanol. The rehydrated tissue slices were stained for 8–10 min with 2% Hematoxylin solution and then for 1–2 min with 2% eosin solution. The stained tissue slices were dehydrated by ethanol. The dehydrated tissue slices were washed with xylene for three times before being blocked for observation under the microscope.

### Immunohistochemistry

Some tissue slices embedded in wax from each rat were de-waxed using xylene and then gradually re-hydrated using ethanol. The re-hydrated tissue slices were washed with 0.3% H_2_O_2_ methanol solution and then with phosphate buffer. The resulting tissue slices were incubated in 1% bovine serum albumin (BSA) at 20 °C for 15 min. The slices were further incubated with rabbit anti-rat CD31 polyclonal antibodies according to the manufacturer’s instruction. Briefly, the incubation with the primary antibody was conducted at 4 °C for 16 h, and the following incubation with the secondary antibody was conducted at room temperature for 20 min. DAB Horseradish Peroxidase Color Development Kit was used to develop the coloring and Hematoxylin was used to stain the cell Nuclei. The negative control slices were treated with the same procedure mentioned above except that these samples were incubated with PBS instead of the primary antibody before being incubated with the secondary antibody. The stained tissue slices were dehydrated using ethanol. The dehydrated tissue slices were washed with xylene for three times before being blocked for observation under the microscope. Three different visual fields were randomly selected for each immunohistochemical slice under the magnification of 400× to count positive expression of blood vessels.

### Western blot

Some harvested femoral heads were ground in a grinding mortar in the presence of liquid N_2_. After the liquid N_2_ evaporated from the mortar, 300 μL single detergent lysate (containing 3 μL PMSF) was added for further grinding at 4 °C for 30 min. The supernatant was sampled and stored at − 80 °C after the lysate was centrifuged at 12,000 rpm for 10 min at 4 °C. Electrophoresis was performed for the collected supernatant samples using acrylamide gel electrophoresis (PAGE) gels in 15% PAGE gel electrophoresis. After the electrophoresis, the resulting PAGE gels were harvested and trimmed to strips according to the Marker. After the gel strips were washed with distilled water, wet transfer method was used for transferring separated proteins to PVDF transfer membranes. The resulting PVDF membranes were blocked through incubation in TBST containing 5% skim milk powder at room temperature for 2 h, followed by incubation with the primary antibodies at 4 °C for overnight and then the secondary antibodies at room temperature for 1 h. TBST was used to wash the PVDF membranes three times (10 min each) before the PVDF membranes were incubated with BeyoECL Plus kit for 5 min. After the fluorescent bands became obvious, the excess substrate solution was blotted using filter paper. The PVDF membranes were covered with plastic wrap and then pressed with X-ray film before detection and visualization.

### Statistical analysis

All numerical data are presented as mean ± standard deviation. Statistical analyses were performed with PASW Statistics for Windows18.0 (SPSS Inc., Chicago, IL), and differences between groups were tested with one-way ANOVA followed by Post hoc LSD method. A *P* value of < 0.05 indicates a statistically significant difference.

## Results

### Observation of HE staining results

**Fig. 1:**
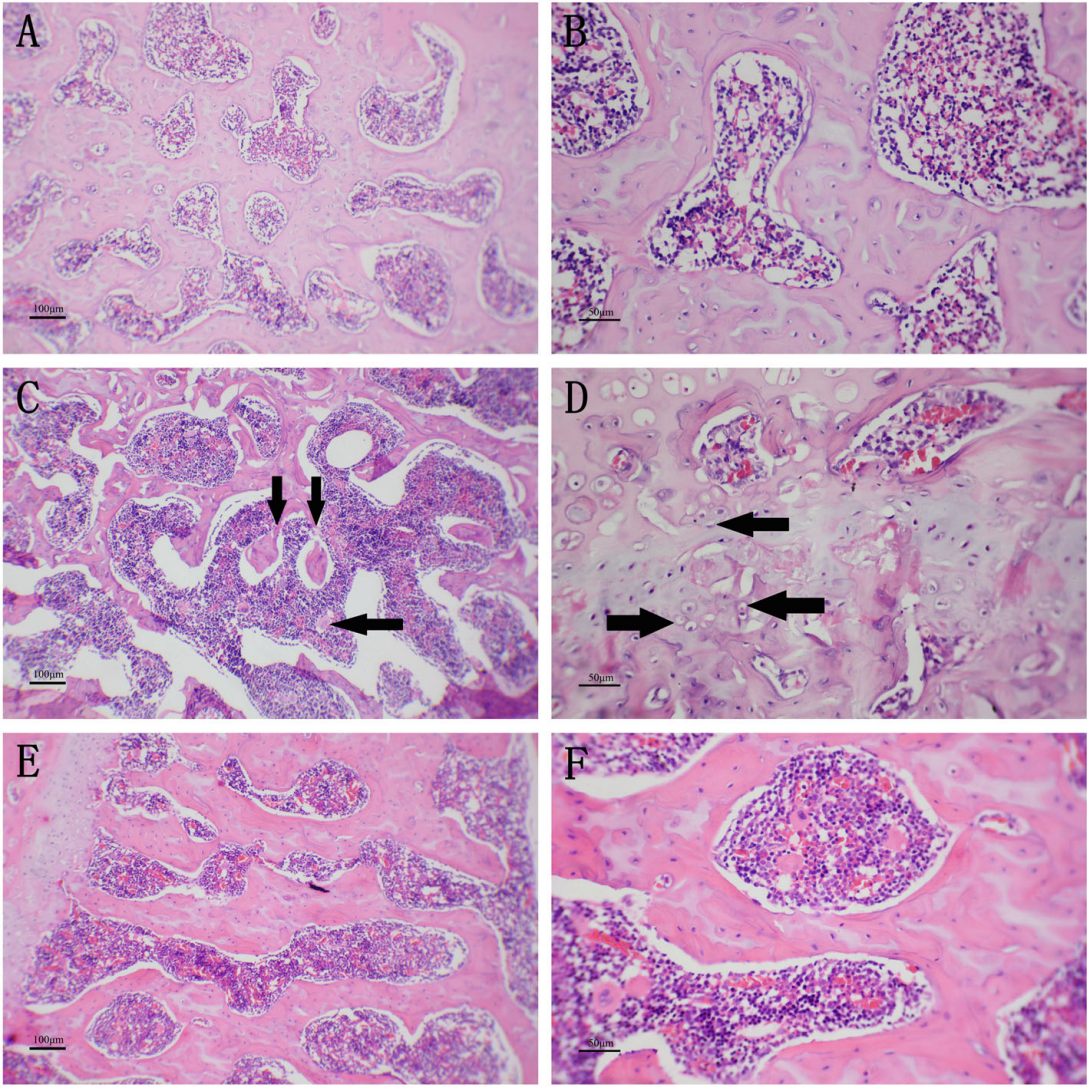
Representative optical images of HE stained femoral head bone slices from rats in negative control group (**a** and **b**), disease group (**c** and **d**), and EPO group (**e** and **f**). Magnification 100× for (**a**, **c** and **e**) (scale bars 100 μm), and 250× for (**b**, **d**, and **f**) (scale bars 50 μm)

Figure 1 showed the representative optical images of HE stained femoral head bone slices in the negative control group (group A), disease group (group B), and EPO group (group C). Group A: an integral circular or oval arched structure and high connectivity without osteoclasts, narrowed bone trabeculae, or fractures was observed in bony trabeculae of Fig. 1a and b. Group B: on contrast, bony trabeculae were obviously sparse, narrowed and fractured with decreased connectivity (Fig. 1c and d). The arch structure partially disappeared and became irregular in shape (marked with an arrow in Fig. 1c). Some cell nuclei had shrunken, dissolved or disappeared (marked with an arrow in Fig. 1d), while more osteoclasts were present. Group C: Fig. 1e and f clearly showed that there were significant improvements observed on the rats from the group C as compared with the group B (Fig. 1e and f). The bony trabeculae were relatively regular in shape. The connectivity was markedly superior to that in group B. Some bony trabeculae had become coarse. The fracture rate was obviously decreased, while connection and repair had occurred in the defects.

### Immunohistochemical expression of PECAM-1

**Fig. 2:**
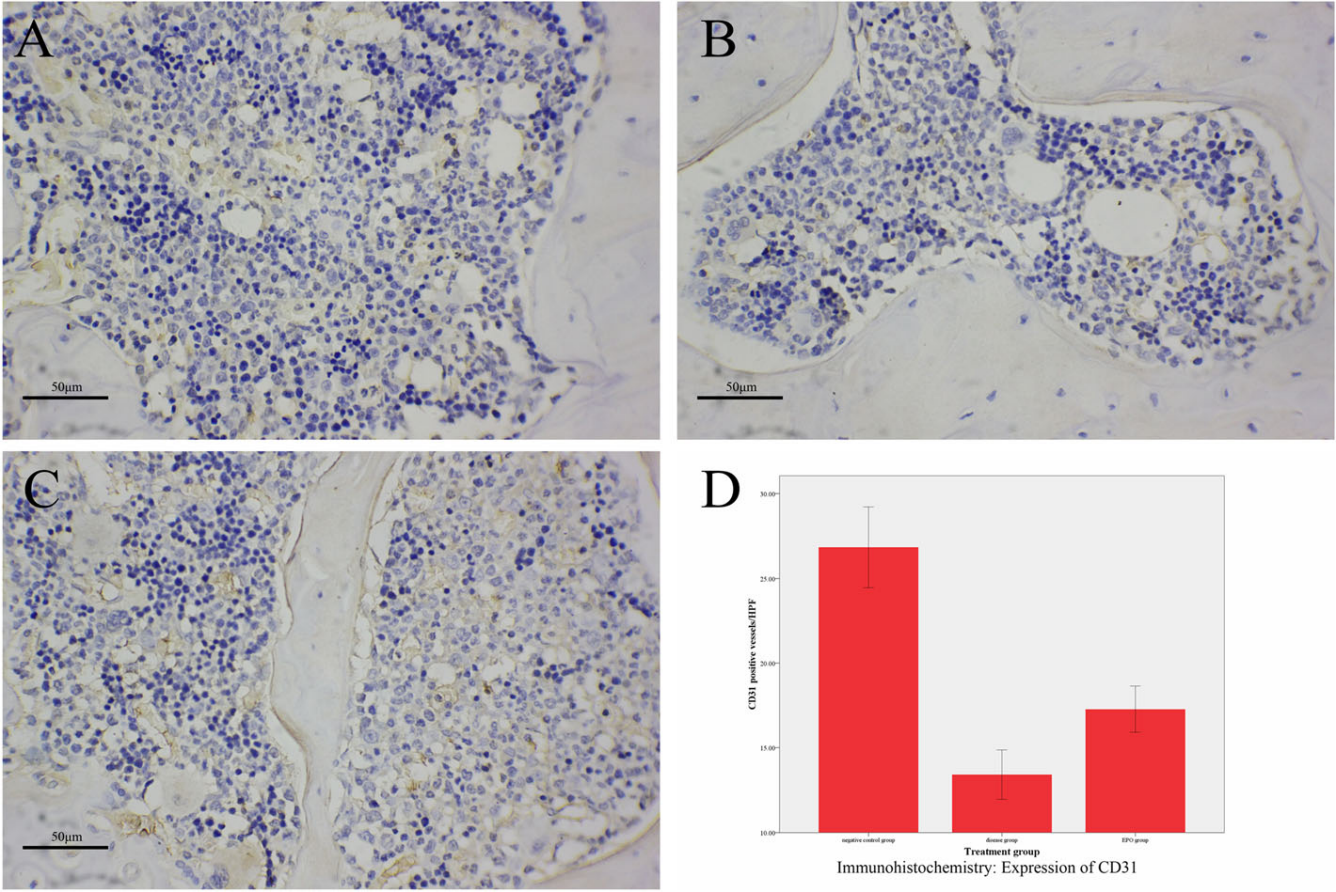
Representative optical images of immunohistochemical femoral head bone slice from rats (**a**, **b** and **c**) in negative control group (**a**), disease group (**b**), and EPO group (**c**); and the expression of CD31 in the femoral head bone determined from immunohistochemistry (**d**). Scale bar: 50 μm. * for *p* < 0.05, and ** for *p* < 0.01

Figure 2a-c showed the representative images of immunohistochemical slices for rats in group A, B and C. Immunohistochemical staining for PECAM-1 was clearly and selectively present in the blood vessel endothelial cells. Positivity was indicated by the presence of yellow particles. In group A, PECAM-1expression was strong, and the blood supply of the femoral head was sufficient (Fig. 2a). The rats in group B (Fig. 2b) had statistically significantly lower blood vessel density in the femoral heads than those in the group A (*p* < 0.01, Fig. 2d, Table 1). However, the blood vessel density in the femoral heads of the rats receiving EPO injection was statistically significant higher than those of the group B (*p* < 0.05, Fig. 2c, Table 1).

**Table 1:** Immunohistochemistry results

	Group A	Group B	Group C
counts/HPF ($\overline{x}\pm s$)	26.83 ± 7.02	13.41 ± 4.27	17.28 ± 4.03
*F*	61.397		
*p*	< 0.01		
Multiple Comparison	Group A-B	Group B-C	Group A-C
*p* (LSD)	< 0.01**	0.03*	< 0.01

### Expression of VEGF in western blot

**Fig. 3:**
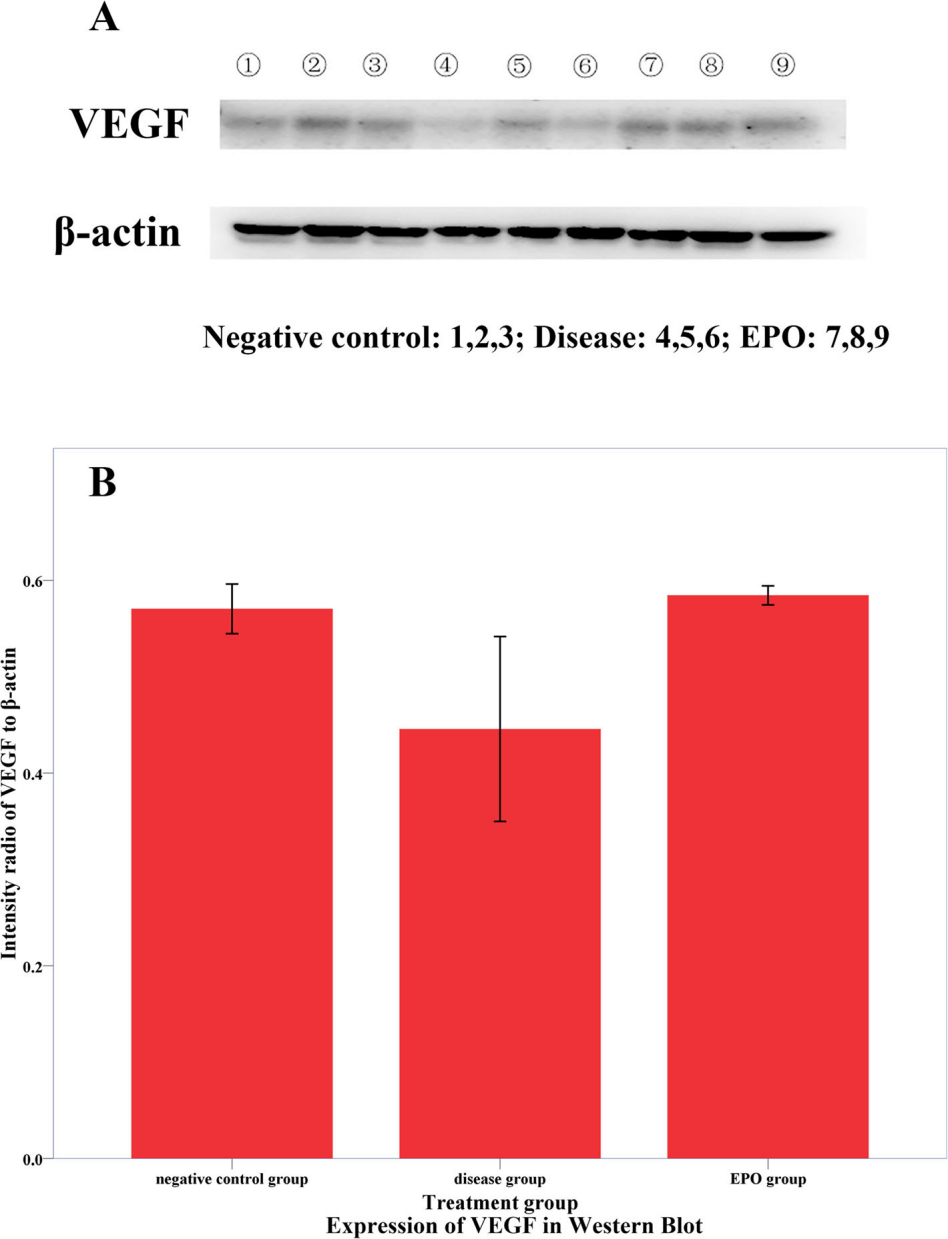
The expression of VEGF in the femoral head bones harvested from all three groups of rats: Western blot images (**a**), and Intensity ratios of VEGF to β-actin shown in A (**b**). * for *p* < 0.05

Figure 3a revealed the expression of VEGF in the femoral heads for all three groups of rats determined by Western blot. The data was quantified and normalized according to the control protein of β-actin (Fig. 3b). The rats in group B had apparently less expression of VEGF in their femoral heads. The injection of methylprednisolone at 20 mg/kg bodyweight caused a statistically significant decrease on the secretion of VEGF in the Group B as compared with the group A (*p* < 0.05, Table 2). This observed decrease was rescued by the co-administration of rHuEPO at 500 u/d/kg-bodyweight (*p* < 0.05, Table 2). Indeed, the ratio of VEGF/β-actin for group C was slightly larger than that for the control group, although the difference was not statistically significant (*p* > 0.05, Table 2).

**Table 2:** Western blotting results

	Group A	Group B	Group C
VEGF/β-actin ($\overline{x}\pm s$)	0.570 ± 0.022	0.446 ± 0.048	0.584 ± 0.005
*F*	7.025		
*p*	0.027		
Multiple Comparison	A-B	B-C	A-C
*p* (LSD)	0.022	0.014	0.742

## Discussion

In this study, we successfully induced ANFH in the rats of disease group by intramuscularly injecting methylprednisolone. Obvious cell apoptosis and avascular necrosis were observed in the HE stained slices of disease group [21, 22], while results of EPO group resembled that of the negative control group.

Sufficient blood supply is reported critical for bone regeneration and skeletal tissue engineering [23]. Some scholars have revealed two main mechanisms, angiogenesis and vasculogenesis responsible for the formation of new blood vessels, also known as neovascularization [23]. Regarding EPO, both of these mechanisms might play roles in the present studies, which could promote bone regeneration via an improved microenvironment and nutrient supply [24]. In this study, the simultaneous injection of EPO clearly slowed down the progress of ANFH. There was evidenced with the observation on HE stained slices of femoral heads in the EPO group of rats. PECAM-1 is highly expressed in vascular endothelial cells, which was used in the immunohistochemical study to define the blood vessel density. The results revealed that the blood vessel density in the femoral heads of the disease group was significantly lower than those in the EPO group, while the results were similar in the control group and the EPO group. For disruption of bone blood supply and essential nutrient supply are the direct cause of femoral head necrosis [11], the effect could be inferred that EPO promotes neovascularization. Besides neovascularization, other mechanisms and signaling pathways may be involved in the protective function of EPO. Shiozawa et al. [25] reported EPO promoting the production of BMPs in hematopoietic stem cells by activating the Jak-Stat signaling pathways as well as enhancing bone formation by activating mesenchymal cells to osteoblasts. Kim [26] found that EPO increased the osteoclast numbers and decreased the bone resorption activity in model by increasing the expression of NFATc1 while decreasing cathepsin K expression in mTOR signaling pathway. Hu et al. [27] discovered EPO could inhibit p38MAPK, reduce the TNF-α level, alleviate the inflammatory injury, and alleviate inhibit apoptosis.

Reducing the cell apoptosis, restoring the bone blood supply and nutrient supply are essential in successful treatment or management of ANFH [28, 29]. VEGF is an angiogenic factor which has a critical role in bone formation and bone healing [30]. In addition to angiogenesis, studies indicated that VEGF and endothelial cells induces osteogenic differentiation of bone marrow-derived mesenchymal stem cells [31]. In this work, the disease group had apparently less expression of VEGF in their femoral heads, which matches other researchers’ results. Li et al.[10] found that dexamethasone, could reduce the synthesis of VEGF protein by inhibiting the bone marrow multipotent cell. Recent studies have proved that VEGF may play a role in bone formation and bone repair [32]. Based on the reports of this study and other scholars, we speculate the possible mechanisms including: (1) EPO increases VEGF expressions and formation of blood vessels, which leads to promoting bone formation and osteoblast differentiation; (2) Besides, EPO might perform osteogenic action and inhibit apoptosis mediated via multiple signaling pathway, which is based on reports of other scholars and remains to be further researched.

Reports questioning the promoting functional effects of EPO in bone have also been published [33]. Despite the controversy of how EPO affects bone tissue, most scholars hold the view that the effect of EPO on bone tissue is site specific and dose-dependent [34]. So in this research, 20 IU/mL EPO was applied, which was demonstrated effective both in vivo and vitro [35].

## Conclusions

In summary, this study suggested the use of rHuEPO at the same time with steroid has achieved a certain precaution effect for steroid-induced ANFH. However, its long-term effect and preventive mechanism require further research and observation.

## Abbreviations

ANFH: Avascular Necrosis of the Femoral Head
BMP: Bone Morphogenetic Protein
DAB: Diaminobenzidine
EPO: Erythropoietin
GC: Glucocorticoid
HE staining: Hematoxylin-Eosin Staining
HPF: High power Field
JAK-STAT: The Janus Kinase-Signal Transducer And Activator of Transcription
LSD: Least—Significant Difference
mTOR: The Mammalian Target of Rapamycin
NFATC1: Nuclear Factor of Activated T Cells C1
one-way ANOVA: One-way Analysis of Variance
PAGE: Polyacrylamide Gel Electrophoresis
PBS: Phosphate Buffered Saline
PECAM-1(also CD31): Platelet Endothelial Cell Adhesion Molecule
PVDF: Polyvinylidene Fluoride
rHuEPO: Recombinant Human Erythropoietin
TNF-α: Tumor Necrosis Factor α
VEGF: Vascular Endothelial Growth Factor
MAPK: Mitogen Activated Protein Kinases
